# Power Disturbance Monitoring through Techniques for Novelty Detection on Wind Power and Photovoltaic Generation

**DOI:** 10.3390/s23062908

**Published:** 2023-03-07

**Authors:** Artvin Darien Gonzalez-Abreu, Roque Alfredo Osornio-Rios, David Alejandro Elvira-Ortiz, Arturo Yosimar Jaen-Cuellar, Miguel Delgado-Prieto, Jose Alfonso Antonino-Daviu

**Affiliations:** 1 CA Mecatrónica, Facultad de Ingeniería, Universidad Autónoma de Querétaro, Av. Río Moctezuma 249, Querétaro 76807, Mexico; 2 MCIA Research Center Department of Electronic Engineering, Technical University of Catalonia (UPC), 08034 Barcelona, Spain; 3 Instituto Tecnológico de la Energía, Universitat Politècnica de València (UPV), Camino de Vera s/n, 46022 Valencia, Spain

**Keywords:** novelty detection, machine learning, power quality disturbance, wind generation, photovoltaic generation

## Abstract

Novelty detection is a statistical method that verifies new or unknown data, determines whether these data are inliers (within the norm) or outliers (outside the norm), and can be used, for example, in developing classification strategies in machine learning systems for industrial applications. To this end, two types of energy that have evolved over time are solar photovoltaic and wind power generation. Some organizations around the world have developed energy quality standards to avoid known electric disturbances; however, their detection is still a challenge. In this work, several techniques for novelty detection are implemented to detect different electric anomalies (disturbances), which are k-nearest neighbors, Gaussian mixture models, one-class support vector machines, self-organizing maps, stacked autoencoders, and isolation forests. These techniques are applied to signals from real power quality environments of renewable energy systems such as solar photovoltaic and wind power generation. The power disturbances that will be analyzed are considered in the standard IEEE-1159, such as sag, oscillatory transient, flicker, and a condition outside the standard attributed to meteorological conditions. The contribution of the work consists of the development of a methodology based on six techniques for novelty detection of power disturbances, under known and unknown conditions, over real signals in the power quality assessment. The merit of the methodology is a set of techniques that allow to obtain the best performance of each one under different conditions, which constitutes an important contribution to the renewable energy systems.

## 1. Introduction

In present times, the demographic growth has brought a noticeable increase in the power energy consumption around the globe, which causes its overproduction for the purpose of covering the final user demands [1]. Regarding this situation, the conventional ways to produce power energy, by taking fossil fuels and converting them to electric energy, has incited negative environmental effects boosting climate change [2]. For that reason, several countries have decided to improve the production of energy through alternative ways better known as renewable generation systems (RGS), considering mainly wind power generation (WPG) [3] and solar photovoltaic generation (SPG) [4]. However, an important aspect to be considered in both cases, conventional and renewable generation systems, is the quality of the energy produced, since it will be used for feeding the final loads such as electric machines, power devices, and electronic devices [5]. Good power quality is essential because proper operation, functionality, and lifespan of the equipment connected to the electric grid will strongly depend on it [6]. Therefore, the analysis of the power quality is essential, and there exist different methodologies that have addressed this topic, such as heuristic techniques [7], model-based techniques [8], data-driven techniques [9], and deep-learning techniques [10]. However, the characteristics of the evolving and emerging technologies add complexity to the power quality analysis, since they are not only affected by the problems in the grid but also generate some of these problems, i.e., the development of new strategies becomes necessary to address these problems [11]. Recently, other methodologies such as the approaches for novelty detection (ND) have proven their effectiveness in industrial applications not related to the power quality study, and, in this sense, it would be interesting to demonstrate how they could be used to provide some solutions to this issue.

In general, the ND encompasses several techniques that can be classified in the following general categorizations [12,13]:

- Probabilistic-based. Here, the data distribution can be thresholded, which in turn can be useful for defining in the data space limits or boundaries of normality. With this information, it is possible to determine if a single sample belongs to the same distribution or not. For such a task, it would be necessary to estimate probability density functions, for example, Gaussian mixture model (GMM) [14].
- Distance-based. These approaches make use of well-defined distance metrics that help defining the similarity between two samples (data points) from a data set, for instance, k-nearest neighbor (kNN) [15] and isolation forest (IF) [16].
- Domain-based. These approaches need the generation or definition of boundaries according to the form of the training data set. It means that they describe the domain of target classes being insensible to the sampling and density, such as one-class support vector machines (OC-SVM) [17].
- Reconstruction-based. These methodologies are widely used in applications that require classification tasks and regression purposes. Normally, these techniques perform data modeling automatically without supervision, and the estimation or prediction is used to obtain a performance metric. That metric is the difference between a test vector and an output vector, better known as the reconstruction error. Thus, this metric is related as a novelty score. For example, there are stacked auto-encoders (SAE) [18] and self-organizing maps (SOM) [19].
- Theoretic information-based. For these approaches, the entire data set is used for computing specific metrics, such as entropies, energies, forms, and moments. With this information, the novelty means alterations in these values compared with normal data sets, such as time–frequency ridge estimation (TFRE) [20,21], degree of cyclostationarity (DCS) demodulation [22], and entropy measure-based methods (EMBM) [23].

There exist several approaches that belong to the aforementioned categorization. However, only one or two techniques, presented as examples, will be taken for the analysis. Therefore, the adopted techniques can be considered a good representation of each category according to the reviews reported in the literature [12,13]. Additionally, according to the analysis of these reviews, the selected techniques are most frequently used, because their implementation is relatively easy and information of different applications is readily available. Therefore, Table 1 presents a brief analysis and discussion of such techniques used in different applications.

Now, as mentioned, it is very important to monitor the power sources that feed the industrial equipment, because in conventional networks and in renewable generation systems, there are elements connected to the grid that generate problems affecting the power quality (PQ). It is worth mentioning that all the equipment and devices are affected by some events or anomalies present in the power grids when they are connected to it. In addition, these devices are, in great part, the causes of these problems [36]. Now, these anomalies are better known as power quality disturbances (PQDs), which are defined, in general terms, as any deviation from the behavior of a pure sinusoidal waveform with a specified amplitude, frequency, and phase [37]. Thus, the PQDs are catalogued according to their features such as duration, frequency value, amplitude variation, and behavior. For that reason, there exist two well-known standards that define and classify these PQDs such as the case of the IEEE-1159 standard [38], where the classification of the most common PQDs are transients, short duration variations, long duration variations, voltage imbalance, waveform distortion, voltage fluctuations, and power frequency variations, summarized in the Table 2. The other important standard is the IEC 61000-4-30 [39], which classifies the power quality parameters in power frequency, magnitude of the supply voltage, flicker, supply voltage dips and swells, voltage interruptions, transient voltages, supply voltage unbalance, voltage harmonics, voltage interharmonics, main signaling voltage on the supply voltage, rapid voltage changes, and measurement of under-deviation and over-deviation parameters, summarized in Table 3. In the IEC61000-4-30 the subclasses advanced (A), surveys (S), and basic (B) are defined for the measured parameters, according to the final application, for precise measurements and power quality assessments, and for instruments with obsolete design, respectively. It is important to clarify that from all the possible PQDs, only some of them will be adopted for the analysis in this work, because the real signals from the renewable systems only present some of these disturbances. Additionally, other anomalies, outside the standards, will be included in the analysis because they are inherent in the renewable generation systems, such as climatic conditions.

Regarding the approaches that address the issue of power quality analysis (PQA) through techniques for ND, there are few works that can be discussed. For instance, in [40], an online method for detecting events with low quality through phasor measurement units and IF is proposed. However, they focus on anomalies such as the noise in the voltage signals and oscillations with growing magnitude. Now, some research directly tackles the PQD classification through hybridizing the kNN with a fully convolutional Siamese network for voltage signals with small number of samples [41]. In the meantime, [42] combined the Riesz transform (RT) of two dimensions, for feature extraction, the multi-objective grey wolf optimizer (MOGWO) together with the kNN, and for selecting and learning features, thus classifying the PQD. On the other hand, some works have used the SVM for detecting and classifying electric disturbances. For example, in [43]. Firstly, the frequency components in the signal through the fast Fourier transform (FFT) are computed, then a set of adaptive filters extracts mono-frequency components of the distorted signal, posteriorly six time-domain features, are computed and fed to multiclass SVM for classification. In other example [44], a combination of a histogram of oriented gradients (HOG) implemented in four steps (gradients computing, binning orientation, concatenation, and normalization) and the SVM are used for distinguishing PQDs. In the same field, a five-step method is presented in [45], where it is first simulated a set of PQDs; second, the variational mode decomposition (VMD) decomposes the signal into instinct mode functions (IMFs); third, time-domain features are extracted from the IMFs; fourth, a heuristic selection is made through the permutation entropy and the fisher score algorithms, and fifth, the multiclass SVM classifies the disturbances. At last, but not least, the research presented in [46] describes a methodology for classifying PQDs in WPG implemented in five steps: generation of a synthetic database for training purposes, multi-domain features estimation, feature reduction through genetic algorithms (GA) and principal component analysis, modeling the power disturbances with SOM, and classifying them through SoftMax. It is worth mentioning that such work focuses only on one technique for ND, which is SOM. but for the other type of renewable generation system, which is WPG, it is worth mentioning that they optimize the feature selection process.

According to the revision of works reported in the literature, it was found that the power quality assessment is generally addressed from the point of view of signal decomposition, for further spectral analysis, feature extraction, and machine learning classifiers. However, few research has been developed from the point of view of the techniques for novelty detection on applications related with the power quality of the renewable energy generation systems, mainly the solar photovoltaic and the wind power generation. Additionally, some restrictions require to be overcome several challenges, such as power disturbances variability, sudden changes, and intrinsic conditions. The existing work, consider faults detection in the parts of the mechanical systems. However, the topic of the power quality is also an interesting field since the evolving technology causes complex problems in the power grid that affect all the devices connected to it, and classical methodologies and measurement processes may find difficulties to provide a solution.

The contribution of this work is the development of a methodology that implements six techniques of ND for detecting and classifying PQDs over datasets of real signals acquired from renewable energy generation systems (REGS), specifically SPG and WPG. The proposed approach will consider PQDs under different scenarios based on the standards IEEE-1159 and IEC 61000-4-30, such as sags, oscillatory transients, and flickers. In addition, there will be considered combinations of these PQDs and abnormal behaviors that are not deemed in the standards, such as meteorological conditions that affect the energy generation process. Such scenarios will include the appearance of the PQDs and their combinations in a dynamic that contemplates two situations: known conditions and novelty condition. The implementation of the techniques for ND comprises k-NN, OC-SVM, GMM, SAE, IF, and SOM, and their algorithms are tested and evaluated for the disturbances proposed. The obtained results demonstrate the effectiveness of every technique emphasizing the usefulness and potential according to the problem to be solved. Therefore, for each of the techniques, it will provide the performance reached, highlighting their adequateness for detecting the power disturbances adopted (sag, oscillatory transient, and flicker), other abnormal condition (meteorological affectation), and which of them are limited in this task.

## 2. Theoretical Foundations

### 2.1. Stacked Autoencoder (SAE)

**Figure 1:**
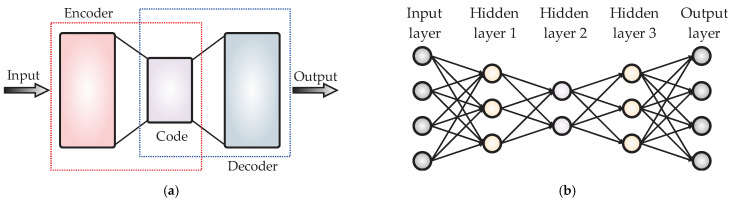
Typical structures of the (**a**) AE and (**b**) SAE.

Autoencoders (AE) are types of unsupervised neural networks with the objective of replicating the input data to the output, as goals of the feature extraction and the dimensionality reduction of the original data [18]. In the structure of the AE, as shown in Figure 1a, the “encoder” block consists of two layers of neurons: the first one having number of neurons equal to the number of input data and the second one having a final dimension, i.e., the data that will be reduced to. Thus, the encoder outputs are the features from the input data in a lower dimension. Based on its part, the “decoder” block also has two layers of neurons: the first one with a size of the reduced representation made by the encoder and the second one with a dimension of the original data. Then, the decoder reconstructs the original data by means of the reduced representation. When a single AE is insufficient for reducing data, a solution is to use several AE integrating different reduction stages, as shown in Figure 1b, and these structures are known as stacked AE (SAE). The SAE requires the minimization of a cost function, *J*_(*x*)_, as expressed in (1).

(1) $$J_{\left(x\right)}=L\left(x,\hat{x}\right)+\Omega \left(x\right)$$

where $L\left(x,\hat{x}\right)$ is the loss function (normally the mean square error); $\hat{x}$ is the output; *x* is the input; and Ω(x) is a penalization term.

### 2.2. One-Class Support Vector Machine

Support vector machines (SVMs) are a set of algorithms that allow to create a non-lineal decision region through the data projection by using a non-lineal function into a high-dimension space [17]. Thus, the data points that cannot be separated by a straight line in their original space *I* are transferred to a space *F* where they can be separated by means of a hyperplane, allowing the identification of various classes. When the hyperplane is projected back into the original space *I*, it will have the shape of a nonlinear curve. The distance from the closest point of each class to the hyperplane is of the same magnitude but looking at the maximum separation between the classes. Therefore, the OC-SVM separates the data points from the origin, inside the feature space *F*, and maximizes the distance between the hyperplane and the origin. This yields a binary function that captures the regions of the input space where lies the probability density of the data. This function returns a positive score in a region that captures the data points for training and a negative score in any other region. The objective function of SVM is observed in (2), subject to (3), and the minimizing function of the OC-SVM (such a s the conventional SVM) can be observed in (4).

(2) $$min\left\{\frac{{\left\|w\right\|}^{2}}{2}+C\overset{n}{\underset{i=1}{\sum }}\xi_{i}\right\}$$

(3) $$y_{i}\left(w^{T}\phi \left(x_{i}\right)+b\right)\geq 1-\xi_{i}$$

(4) $$min\left\{\frac{{\left\|w\right\|}^{2}}{2}+\frac{1}{Nv}\overset{n}{\underset{i=1}{\sum }}\xi_{i}-\rho \right\}$$

where *w* is the normal vector to the hyperplane; $\xi_{i}$ denotes the distance to the margin; C is a regularization parameter; $\phi \left(x_{i}\right)$, corresponds to the input vector of transforming space; *b* is a bias; $y_{i}$ is the value of the *i-th* sample; n is the number of samples; Nv is the parameter that characterizes the solution; and ρ is the interval to the hyperplane.

### 2.3. k-Nearest Neighbors

k-nearest neighbor (kNN) is a supervised algorithm used as a learning classifier based on the data proximity that performs clustering [15]. kNN works by assuming that similar points can be found near to each other, being the objective of the identification of the nearest neighbors from a given query point, then a class label is assigned to it. A class label is assigned to the label that occurs with more frequency around a determined point. Then, the algorithm classifies the data in the corresponding group, depending on whether it has *k* neighbors closer to one group or another. The distances are calculated from one point to the other existent points and arranged in ascendent order. Consequently, the group will be the most frequent with lower distances. There exist several ways to compute the distances, such as the Euclidian distance (5) and the Manhattan distance (6).

(5) $$d\left(x,y\right)=\sqrt{\sum_{i=1}^{k}{\left(x_{i}-y_{i}\right)}^{2}}$$

(6) $$d\left(x,y\right)=\sum_{i=1}^{k}\left|x_{i}-y_{i}\right|$$

where, d(x,y) corresponds to the distance between two pints, $x_{i}$ is the *i-th* position in the plane horizontally, $y_{i}$ is the *i-th* position in the plane vertically, and i=1, 2, 3,…,k.

### 2.4. Gaussian Mixtrure Model

The Gaussian mixture model (GMM) is a function composed of several Gaussians, each identified by k∈{1,2,3,…,K}, where K is the number of clusters in the dataset [14]. Every Gaussian function k of the mixture is composed of the following parameters:

- A mean μ defining its center.
- A covariance Σ specifying its width.
- A mixture probability π that defines how large or small the Gaussian function will be.

The mixing coefficients themselves are probabilities and must satisfy the condition of (7).

(7) $$\sum_{k=1}^{k}\pi_{k}=1$$

### 2.5. Self-Organizing Maps

The self-organizing maps (SOMs) are unsupervised learning schemes that provide a way to represent multidimensional numerical data in lower-dimensional vector spaces, typically 2D/3D [19]. Generally, the SOM comprises an architecture of two layers: a layer of learning nodes, which will ultimately contain the information about the resulting representation, and the layer of input nodes, which will serve as feeders for the learning nodes by means of the original vectors (data) during the training process. The first layer nodes are connected to all the elements of the second layer and have weights that can be changed by learning the information from the input nodes. The purpose of the algorithm will be to find the appropriate weights of the connections between both layers to provide a representation of the input data in the geometric structure of the learning nodes. Because there is no objective function to approximate, the SOM attempts to provide some representatives of weights in the learning nodes in such a way that a double objective is verified:

1. Nearby learning nodes have nearby weight vectors.
2. Each input data has some close representative among the learning nodes (in the sense that there is some learning node whose weight vector resembles the input data).

To do this, the SOM starts from an initial weight distribution (usually random), then tends to approximate a stable weight distribution. After stabilization (convergence of the algorithm), each of these learning nodes (and the nodes close to it) groups the original data that most resemble it so that the entire network becomes a topological classification tool.

### 2.6. Isolation Forest

**Figure 2:**
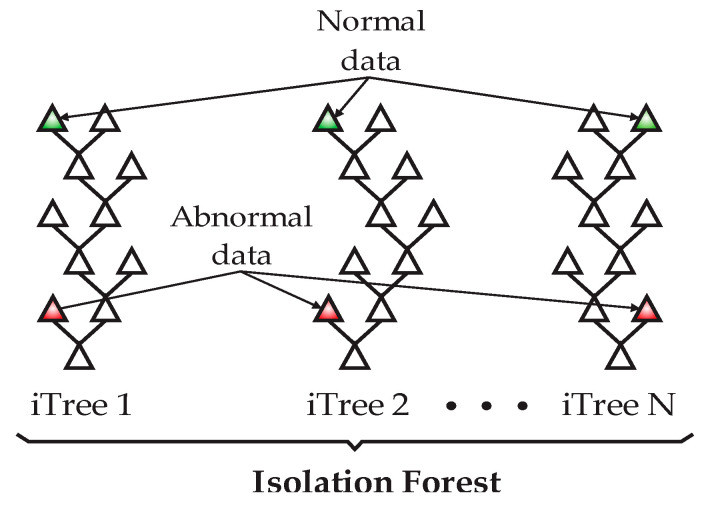
Typical structures of the isolation forest.

The isolation forest (IF) is an unsupervised algorithm based on decision trees having the function of identifying abnormal data and isolating them from the entire dataset [16]. The IF isolates observations by randomly selecting a feature and randomly selecting a division between the maximum and minimum values of it. A division is a tree structure (iTree), and the number of partitions for isolating a sample equals the length of the path from the root to the end point of the branch, as shown in Figure 2. The length of the branches, together with the isolation forest structure, represents a measure of normality and the decision function. The anomalies or novel data will be represented through shorter paths inside the forest structure.

## 3. Proposed Methodology

**Figure 3:**
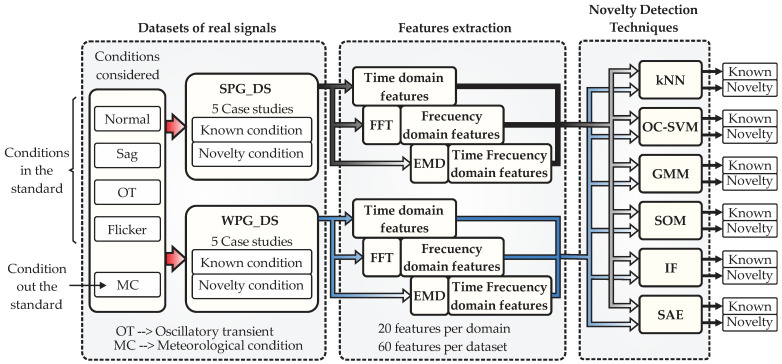
General block diagram of the methodology for implementing the techniques of ND.

In this section, the proposed methodology that implements the six techniques of ND for diagnosing and classifying electric disturbances, considered in the standards IEEE-1159, IEC 61000-4-30, and outside the standards, will be described. The signals with power disturbances analyzed correspond to two types of renewable energy generation systems, SPG and WPG. Next, Figure 3 presents the general block diagram of the proposed methodology, as it can be observed, three main blocks integrate the approach: (i) datasets of real signals, (ii) feature extraction, and (iii) novelty detection techniques. In the following lines each one of these blocks will be described in detail.

From Figure 3, the first block refers to all related data used for evaluating the performance of the techniques for ND. This block comprises two datasets: the first one corresponds to an application of energy generation by means of solar photovoltaic panels (SPG_DS), obtained from a photovoltaic park, and the second one is also related to energy generation but through wind turbine systems (WPG_DS), obtained from a wind farm. For each dataset, five case studies will be considered for the evaluation, and such cases imply that the analyzed signals will divide the information into two conditions: known condition and novelty condition. The known condition are those signals from which the technique for ND will be trained by learning the extracted features, which means that the technique will learn the behavior of the signals defined as known condition. The novelty condition are those signals from which the technique for ND have any previous information or training. In other words, these signals will be fed to the technique to be considered novelty. To this end, the signals considered for being processed include the normal condition and power disturbances addressed by the standards IEEE-1159 and IEC 61000-4-30, such as sag, oscillatory transients (OT), and flicker; however, a condition outside the standard is also considered such as a meteorological condition (MC). Here, the MC belongs only to the signals from the SPG_DS, and it refers to climatic variations (cloudy day) that generate incipient changes in the signal amplitude. Finally, the datasets are evaluated individually, and the signals are processed in the next block.

In the second block, the feature extraction from the signals of the datasets (SPG_DAS and WPG_DS) is performed. For example, the signals from the SPG_DAS with the power disturbances are sent to this block, then time-domain features, frequency-domain features, and time–frequency domain features are computed. The time-domain features are extracted directly from the input signals, but the extraction of the frequency-domain features needs firstly the application of the FFT. Similarly, with the aim of extracting the time-frequency domain features, first, it is necessary to apply a space transformation through the empirical mode decomposition (EMD) by using the first instinct mode function (IMF). A total of 20 statistical and non-statistical features are computed, which comprise indicators such as mean, maximum value, root mean square (RMS), square root mean (SMR), standard deviation, variance, RMS with shape factor, SMR with shape factor, crest factor, latitude factor, impulse factor, skewness, kurtosis, 5th moment, 6th moment, energy, entropy, range, form factor, and log energy entropy. These features are selected because they can provide data information not directly visible from the signals (even after a domain transformation), for instance, they can give central tendencies, dispersions, patterns, profiles, distributions, geometry, asymmetries, form, energy, entropies, among others. In addition, they have demonstrated their effectiveness in other applications, such as in monitoring systems [47], and they can be easily implemented and computed through the equations observed in Table A1 of Appendix A. It is worth mentioning that, with these features, the provided information of the signal was sufficient for this work. However, if the final application is characterized by other type of signals such as acoustic ones, there can be added features related to these signals. In other words, the more features can be obtained from the signals, the more information can be used by the techniques of ND, however, with a caution because in contrast much of the information is not useful; instead, it will depend on the application and the technique implemented. Therefore, considering the 3 domains (time, frequency, and time-frequency) and the 20 statistical and non-statistical features, a total of 60 features describe the signal with power disturbances from the SPG_DS. Additionally, these 60 features are extracted for each of the five case studies considered in the dataset. The abovementioned procedure is repeated similarly over the signals from the WPG_DS. Thus, the features from the datasets per case study are fed to the ND technique block for further processing.

In the third, and last block, a set of six different techniques comprise the novelty detection technique stage. The techniques implemented in this block include kNN, OC-SVM, GMM, SOM, IF, and SAE. The features extracted from the datasets are fed one by one, case study per case study, following the dynamic described next. To exemplify, the analysis of the signals from the SPG_DS will be described. Thus, the analysis begins by feeding the 60 extracted features of the first case study that corresponds to the subset defined as known condition to the set of techniques for ND, and with this information, the six techniques are trained. It must be remembered that these features consider some PQDs and the six techniques learn the patterns of such anomalies. Posteriorly, the six techniques are used for detecting novel conditions as follows. Once the techniques for ND are trained, they are fed with input vectors that have features of both types: known and novelty conditions. The purpose is that the techniques for ND indicate when the input features belong to the known conditions or to the novelty. This way, the rest of the case studies are analyzed similarly, and in turn, all the same procedure is repeated for the signals from the WPG_DS. It is worth mentioning that some case studies consider combinations of PQD and MC for the training of the techniques. Therefore, the outputs of the proposed structure of Figure 2 are the classifications label “known” and “novelty”, which can be considered a semaphore that indicates if a signal behaves as usual or if an unknown condition occurs. Finally, when an unknown condition happens, the techniques for ND will indicate it as novelty, later the technique will learn about this situation (through training), assuming it is known. If a new unknown condition appears, one more time, it will be assumed as novelty. This way, the performances of the six ND techniques can be compared.

## 4. Experimental Results

### 4.1. Experimental Setup

As previously mentioned, the data for the methodology comprise two datasets: the first one has the signals that were acquired from a 30-MW photovoltaic park (SPG_DS) and the second one has the signals acquired from a 100-kW wind farm (WPG_DS). All the acquired signals from SPG and WPG work on an operating frequency of 60 Hz and were captured at a sampling frequency of 15.36 kHz. Originally, the amplitude of the signals from the photovoltaic park was of 230 Vrms, and the original amplitude of the signals from the wind farm was of 45 kVrms, but all the magnitudes were normalized yielding the final signal per unit. For each data set and for each one of the case studies, 1000 samples were used for training the techniques for ND. For the normal condition, a synthetic signal with white noise added with an amplitude of 60 dB was used. Based on this, in the experimental runs on the SPG_DS, 20 signals were analyzed per case study, 10 for the known condition training and 10 for the novelty detection. In the case of the experimental trials on the WPG_DS, 30 signals were analyzed per case study, 15 for the known conditions training and 15 for the novelty detection. Each one of the 1000 samples considers time windows of 10 cycles from the input signal waveform with the aim of accomplishing the standard requirements for signal processing. Now, the feature extraction comprises not only a total of 20 statistical and non-statistical indicators with the objective of covering aspects that are not easily visible directly from the original data, such as distributions, geometry, tendency, asymmetry, patterns, and profiles, but also indicators of energy and entropy. Since these 20 features are computed from the time domain, frequency domain, and time–frequency domain, a total of 60 features per sample and per case study are used on each dataset. The experimental tests were carried out in a PC laptop Intel (R) Core (TM) i5-6500 CPU running at 3.2 GHz, 16 GB RAM, and NVIDIA Quadro P620 GPU, by using the MATLAB software. Now, the implementation of the six techniques for ND requires the configuration of hyperparameters, which were defined through experimentation. These values are summarized in Table 4.

### 4.2. Results of the Signals from the SPG_DS

In the following lines, the results obtained for the five case studies of the dataset “SPG_DS” will be analyzed by comparing the performances of the six techniques for ND.

#### 4.2.1. SPG_DS 1

In this case study, a signal without PQD (electric disturbance in the standard) and without MC (abnormal condition out the standard), which is considered as normal behavior, is used first as the known condition, and a signal with OT (a PQD) is considered the novelty condition. Therefore, the six techniques for ND are trained by using the extracted features from the normal signal (known). Once the techniques have learned the feature behavior, then the novelty detection is tested by feeding the six techniques with two input vectors: the first contains features of the normal signal and the second contains features of the signal with OT. The obtained results for the six techniques are shown numerically in Table 5.

#### 4.2.2. SPG_DS 2

In the second case study, again, the normal signal is used first as the known condition, but now a signal with MC (meteorological condition out the standard) is considered novelty condition. Similarly, the six techniques for ND are trained by using the extracted features from the normal signal (known). For this case, the novelty detection is tested by feeding all the techniques with two input vectors: the first containing features of the normal signal and the second containing features of the signal with MC. The results for the second case are shown numerically in Table 6.

#### 4.2.3. SPG_DS 3

For the third case study, a combination of the normal signal with the OT disturbance is used now as the known condition. Based on this, the novelty condition remains as a signal with MC. Thus, the six techniques for ND are trained by using the extracted features from the signals of the known condition. The two input vectors for testing the novelty detection are now the known condition (normal and OT) and the signal with MC. The results for the third case are shown numerically in Table 7.

#### 4.2.4. SPG_DS 4

Regarding the fourth case study, similarly as the third case, a combination of the normal signal but with the MC disturbance is used as the known condition. Now, the novelty condition will be OT disturbance. Next, the six techniques for ND are trained by using the features of the known condition. In this case, the two input vectors for validating the novelty detection are now the known condition (normal and MC) and the signal with OT. The results for the fourth case are shown numerically in Table 8.

#### 4.2.5. SPG_DS 5

Finally, in the fifth case study the normal signal is considered again the known condition, but the novelty condition will be the combination of the OT and the MC disturbances. With these specifications, the six techniques for ND are trained by using the features of the known condition. For validating the novelty detection in the fifth case, the two input vectors are the features of the normal signal and the features of the combination of OT and MC. The results for the fifth case are shown numerically in Table 9.

### 4.3. Analysis of the Signals from the WPG_DS

Now, in the next lines, the results obtained for the five case studies of the dataset “WPG_DS” will be analyzed by comparing the performances of the six ND techniques.

#### 4.3.1. WPG_DS 1

The first case study of the WPG_DS considers the normal signal as a known condition and considers the flicker disturbance as novelty condition. This way, the six techniques for ND are trained by using the extracted features from the normal signal. Therefore, the two input vectors for validating the novelty detection of power disturbances are the features from the normal signal and the features from the signal with flicker. The results for the six techniques are summarized in Table 10.

#### 4.3.2. WPG_DS 2

Regarding the second case of the WPG_DS, it uses the normal signal as known condition and uses the sag disturbance as novelty condition. Thus, the training process is carried out by using the extracted features from the normal signal. The two input vectors for validating the novelty detection of power disturbances are the features from the normal signal and the features from the signal with sag. The results for the six techniques are summarized in Table 11.

#### 4.3.3. WPG_DS 3

Now, in the third case study, the combination of the normal signal and a signal with flicker is defined as the known condition. Meanwhile, a signal with sag disturbance is defined as the novelty condition. Here, the training process is carried out by using the extracted features from the combined signal (normal and flicker). The validation of the novelty detection uses the two input vectors: features of combined signal and the features from the signal with sag. The results for the six techniques are summarized in Table 12.

#### 4.3.4. WPG_DS 4

Regarding the fourth case, in a similar way as the third case, the combination of the normal signal but now with a signal having a sag disturbance is defined as the known condition. Meanwhile, a signal with flicker disturbance is defined as the novelty condition. The training process is done through the features from the combined signal (normal and flicker). The validation of the novelty detection uses the two input vectors: features of combined signal and the features from the signal with flicker. The results for the six techniques are summarized in Table 13.

#### 4.3.5. WPG_DS 5

Last, but not least, the fifth case considers again the normal signal as the known condition, but the novelty condition will be the combination of the flicker and the sag disturbances. With these specifications, the six ND techniques are trained by using the features of the known condition. With the purpose of validating the novelty detection in the fifth case, the two input vectors are the features of the normal signal and the features of the combination of flicker and sag. The results for the six techniques are summarized in Table 14.

## 5. Discussion

### 5.1. Discussion of the Results from the SPG_DS

In the next lines, the discussion about the obtained results will be described in detail for the different case studies of the SPG_DS.

#### 5.1.1. SPG_DS 1

It can be observed from Table 5 that the six techniques present in general good performance in classifying the normal signal as known condition, but an excellent performance in detecting the signal with OT as novelty condition. The values observed in Table 5 indicate in detail that the six techniques reach a performance of 100% in classifying the disturbance of OT as a new event. In this table, it is also observed that OC-SVM, kNN, SOM, and IF have a slight error in classifying the normal signal as known condition but reaching a performance above 94.5. Based on this, the worst performances, in this case, are for the SAE and GMM, which reach 88% and 78% in classifying the normal signal as known condition, respectively. The overall performances showed that from the six techniques, five of them achieve above 94% and only the GMM stays slightly under 90%.

#### 5.1.2. SPG_DS 2

From Table 6, we observe good results in general for all the techniques and that all the performances are above 80% in general. More precisely, it is noticed that the six techniques, again, achieve a performance of 100% in classifying as novelty event in MC. Similarly, as the first case, the OC-SVM, kNN, SOM, and IF reach a performance above 94.5% in classifying the normal signal as known condition. Only the SAE and the GMM have a lower performance, of 87% and 81.5%, respectively, in classifying the known condition. In the overall performance, all the techniques achieve above 97.25% except SAE and GMM that keep just above 90%.

#### 5.1.3. SPG_DS 3

The results for this case present remarkable variations between the performances of the techniques for ND, it is clearly observed how the use of a known condition defined by the combination of a PQD with normal behavior yields very differentiated results as compared with previous cases. The numerical values from Table 7 are analyzed in detail. The results of the classification of the known condition are excellent for the SAE, kNN, SOM, and IF techniques, achieving a performance above 93.5%. However, in the same line, the worst results are for the OC-SVM and the GMM, reaching a performance of 79.5% and 66%, respectively. Now, the results of the classification of novelty condition are very interesting for IF that has a low performance of 86.5%, however, particularly, the SOM technique barely reaches 15%. The low performances achieved by SOM and IF could be explained because MCs are incipient changes that could be categorized as known condition and the structures of these techniques are insensible to this information. The general performance is also affected due to the low values reached during the classifications of the known and novelty conditions, leaving SOM as the lowest value with 57%; OC-SVM, GMM, and IF between 82.25% and 89.75%; the SAE and kNN between 94.25% and 97.5%.

#### 5.1.4. SPG_DS 4

The results of the fourth case study show a more constant behavior in the performances of the techniques than the previous case, and the numerical values of Table 8 could provide a precise analysis. Then, from this table, the results obtained for the classification of known condition indicate a good performance for the SAE, kNN, SOM and IF above 90.5%, leaving the OC-SVM and the GMM as the lowest performances with 83.5% and 77%, respectively. On the other hand, the results obtained for the classification of the novelty conditions indicate a performance of 100% for almost all the techniques, except for IF with a low performance of 80%, but SOM has the worst execution achieving a 69%. The overall performances stay for tSAE, OC-SVM, and kNN between 91.75% and 95.75%, for GMM and IF with 88.5% both, and SOM with the lowest value of 77.5%.

#### 5.1.5. SPG_DS 5

In the fifth case study, it is very interesting that the results present consistent values in the performances of the six techniques, which means that they have little variations between them. This asseveration can be verified through the numerical values of Table 9. In this last case, the results achieved for the classification of the known condition are more congruent between the techniques, almost the majority reaches above 94%, and onlySAE and GMM stay just below with 87.5% and 82.5%, respectively. On the other hand, the results of the classification of the novelty condition are excellent with a performance of 100% for all the techniques, except SOM that achieves a good performance of 90%. In this last case, the overall performance for all the techniques stays above 91.25%. The explanation of these results could be justified by the training of the ND techniques through the features of the normal signal, since the novelty events are the MC and the OT disturbances, whih have remarkably different behavior from the normal.

### 5.2. Discussion of the Results from the WPG_DS

Now, the discussion about the obtained results will be described in detail for the different case studies of the WPG_DS.

#### 5.2.1. WPG_DS 1

In this first case of the WPG_DS, the results seem congruent in the total performances for all the techniques except for GMM and SOM, so a detailed analysis by examining the values in Table 10 can give more accurate information. In this first case of the WPG_DS, the results obtained from the six techniques observed from the table reveal actually good results for the classification of known condition above 93.66% for all the cases except SAE and GMM. Although their performance is 87.33% and 80.68%, respectively, these values are not bad. Now, the values of the classification of novelty conditions reveal a perfect execution for five of the six techniques, only SOM reaches 75.01%. In consequence, the total performance for all the techniques stays between 90.33% and 99.18%, the lowest value keeps for SOM with 85.5%.

#### 5.2.2. WPG_DS 2

Similarly, as the first case, the results of the second case study show congruent total performances for three of the six techniques above 97.17%, except for SAE, GMM, and SOM whose performances vary. Therefore, a depth analysis of Table 11 reveals that SAE and GMM achieve performances of 85.01% and 80.33% in classifying the known condition, being the worst values from the six techniques. On the other hand, only the SOM technique just achieves76.67% in the performance for classifying the novelty condition, the rest of the five techniques performs perfect. Nonetheless, the total performance of five of the six techniques stays between 90.17% and 97.68%, with 87.01% being the w orst value reached by SOM.

#### 5.2.3. WPG_DS 3

The results of the third case of the WPG_DS behave similar to those obtained for the third case of the SPG_DS, because they present more variations in the performances for all the techniques. The numerical results summarized in Table 12 are analyzed. From the table, it is observed that the performances of the classification of the known condition are good only for kNN, SOM and IF, achieving values above 91.67%. Next, low performance is achieved by SAE with 87.34%, and the worst values are achieved by GMM and tOC-SVM, with t 74.34% and 69.68%, respectively. Regarding novelty detection, SAE, OC-SVM, and kNN performed perfectly, GMM did good, and SOM and IF reached the lowest values around 80%. The total performances are between 84.83% and 88.18% for OC-SVM, GMM, SOM, and IF, and above t 93.66% for SAE and kNN.

#### 5.2.4. WPG_DS 4

Once again, the results observed for the fifth case study are very variable, a comparable situation as in the fourth case of the first dataset, so a depth analysis of the numerical results is performed through Table 13. From the table, it is observed that for the classification of the known condition, only kNN, SOM, and IF achieve performances above 91%, SAE and GMM with 88% and 83.66%, respectively, and the worst performance is reached by OC-SVM with 64.33%. Now, for novelty detection, SAE, OC-SVM, and kNN have done perfectly, GMM did well, but the worst values are for IF with 47.67%, being the lowest value for SOM with 25.34%. In the total performances, only kNN reaches 95.5%, the rest five techniques stay under the 85.18%, with SOM being the worst with 60.16%.

#### 5.2.5. WPG_DS 5

Finally, in the last case study of WPG_DS, once again, the results show a good and congruent behavior for the total performances achieved by the techniques for ND. Here, Table 14 shows percentages above 91.99% for OC-SVM, kNN, SOM, and IF, but SAE and GMM remains around 80%, which are not bad performances in the classification of the known condition. Meanwhile, for novelty detection, only SOM reaches a performance of 75.34%, which is a low value, and the rest five techniques have done perfectly. Once again, for this particular case, due to the training process through the normal signal, the novelty detection task reaches high performances values when a power disturbance appears. The total performance stays above 90.33%, with SOM being the only technique with 83.84%.

## 6. Conclusions

This work describes a methodology that implements six techniques for novelty detection and the techniques are stacked autoencoder, one-class support vector machines, k-nearest neighbor, Gaussian mixture model, self-organizing maps, and isolation forest. It is worth mentioning that these techniques are implemented under the framework of the power quality assessment deemed by the standards IEEE-1159 and the IEC 61000-4-30 for two important renewable energy systems: solar photovoltaic and wind power generation. In addition, the scheme proposed allows to test the six techniques through a set of features in three different domains (time, frequency, and time–frequency) for an equal analysis of performances, allowing to discern between two conditions: known and novelty. The selected statistical and non-statistical features are sufficiently generalized, and they provide information contained in the signals from the datasets that is not directly visible, for example, data geometry, distribution, central tendency, asymmetries, form, patterns, and energy. Having this in mind, these features provide equality to implementation, no matter the internal structure, principle, or requirements of the technique for novelty detection, it would have appropriate information to be operated. On the other hand, the techniques for novelty detection were implemented to learn a specific behavior of an electric event in the standards (power disturbance) and outside the standards (meteorological condition), and this will be the known condition. Thus, if a condition is not considered in a previously behavior learned by the technique, it must be considered as novelty, and this capability could be used advantageously in an iterative process that learns through the time the features of the new condition, advising when a sudden change happens. These techniques have proven to be adequate for the proposed scenarios, but they could be extended for analyzing other failures, or being optimized or combined with other algorithms as further work.

Now, based on the results obtained from the two datasets analyzed (solar photovoltaic and wind power), several conclusions can be made. For example, it is worth mentioning, at first place, that from the six techniques, k-nearest neighbor was the most congruent algorithm for novelty detection of electric disturbances in both datasets, since for all the cases, this technique achieves overall performances above 94.25%. In addition to this, the congruency of the technique can be noticed because it does not present high variations in the classification of the known and the novelty conditions. In contrast, according to the results from Table 5, Table 6, Table 7, Table 8, Table 9, Table 10, Table 11, Table 12, Table 13 and Table 14, in both datasets, Gaussian mixture model and self-organizing maps can be considered the techniques with the lowest performances. Particularly, Gaussian mixture model, although with low performances for all the cases, remained with low variations on their reached performances for the classifications. Based on this, self-organizing maps performed well for the case studies where only one power disturbance was defined as novelty, or when the known condition is used for training the technique. However, if a combination of a normal signal with a power disturbance is used for training the algorithm, its performance significantly decreases. Regarding the isolation forest algorithm, it can be concluded, based on the table’s summaries, that this technique performs well in most of the cases (above 96.84%), except for those cases where the training process is conducted through the combination of a normal signal with a power disturbance. In such case, it reaches performances between 72.17% and 89.5%. Now, in relation to the stacked autoencoder algorithm, its performances are perfect when classifying novelty conditions, with values of 100%. However, its performance in the classification of the known condition varies between 82.33%, as the lowest value, and 95%, as the highest value, which is a good range. In consequence, all the performances for the stacked autoencoder stay above 91.18%. At last, but not least, one-class support vector machines provided excellent results for the case studies where the algorithm training was through a normal signal, but having drawbacks when the training process was made through the combination of a normal signal with a power disturbance. This was reflected as a performance decrement for the classification of the known conditions achieving values between 64.33% and 100%. Finally, it is concluded that in general, when a combination of power conditions is used for the algorithm training, the worst performance values are obtained when the techniques attempt to classify the known conditions.

## Figures and Tables

**Table 1:** Methodologies for ND according to the general categories.

Category	Technique for ND	Ref.	Application
Probabilistic based	GMM	[24]	Methodology for accurately estimating and testing the number of components in medical images, by model selection criterion, where the image can be, for example, a tomography. It is also defined as a sum of weighted real parts of all log-characteristic functions of the GMM as new convergent function, and it is validated for simulated datasets and medical images in two dimensions of univariate acidity.
		[25]	Estimation of the monthly river flows and their suspended sediments, by using the sample points of the rivers, in such a way that their parameters are adjusted by comparisons of information criteria. The parameters tuned, also known as latent variables, are associated with a mechanism that generates bivariate distributions between two regimes related to the seasons of the hydrological variables. The transitions between the regimes are estimated through the Markov chain, which is a switched regression model that finally predicts the monthly suspended sediment fluxes when the water discharge is known.
Distance based	kNN	[26]	Development of an unsupervised learning strategy for monitoring health under environmental variability based on the combination of the Mahalanobis-squared distance and the one-class kNN rule. This approach trains and tests the datasets by finding as many neighbors as possible, removing the environmental variability conditions, estimating the covariance matrices, and using a generalized model of extreme values distribution.
		[27]	Development of a semi-supervised method of novelty detection in datasets with different characteristics through the reconstruction of the nearest neighbors and the latent-neighbor distances of a given input.
	IF	[28]	Detection of anomalies (outliers and inliers) in data acquired from the manufacturing process of etching. The proposed approach was divided into two main parts: offline stage, for evaluating performance and for updating the approach; online stage, for evaluating the observations at hand.
		[29]	Methodology for detecting railroad station anomalies based on an improvement of IF, Huffman forest. This scheme leverages Huffman encoding to measure abnormalities in diverse railroad scenarios. Thus, the trees of the forest are integrated from the perspective of data points for computing anomalies scores of the instances, considering the local and the global available information.
Domain based	OC-SVM	[30]	An unsupervised classification algorithm that autonomously labels between anomalies and normal traffic in the network for imbalance in classes distributions.
		[31]	Prediction of faults and detection of unknown status in a heating, ventilation, and air conditioning (HVAC) chiller system. Here, the principal component analysis (PCA) is used for remarking the novelties in the chiller, considering the anomalies as faults, the condenser fouling, the condenser water flowing, and the refrigerant leakage as priori unknown conditions.
Reconstruction based	SAE	[32]	Detection of outlier components in data coming from wind turbines (WT). Thus, an unsupervised approach for detecting outliers is developed by combining the stacked denoising auto-encoder (SDAE), for extracting the features from the original data and training purposes, and a density-grid-based method, used for data clustering.
		[33]	Monitoring of tool wear for the continuous cutting process in a milling machine under variable speed conditions. This task is achieved by using the stacked sparse auto-encoder (SSAE), and the measured signals were converted into angle domain stationary signals through the order analysis for performing feature extraction.
	SOM	[34]	Detecting unknown problems in electromechanical systems through multi-fault detection and identification scheme. Three main parts integrate such scheme, the feature extraction in the time domain directly from multiple sensors readings, the data-based model of available operating conditions through SOM, and the incremental learning of new conditions through self-organizing structures.
		[35]	Detecting outliers in business applications for preventing losses and for optimizing the revenue. This is achieved through a set of self-organizing structures that recognize unusual behaviors on data patterns.
Information theoretic based	TFRE	[20]	Monitoring and diagnosis of faults in gears and bearing of rotating machinery through oscillatory components with time-varying amplitudes and frequencies. The component with a sequence of peaks in the time–frequency representation is known as ridge. These ridges enhance the machine condition assessment but require an adequate cost kernel and an adaptive search region detection principle for ensuring the ridges smoothness.
		[21]	Detection of faults in rotating machinery through an automated and adaptive ridge extraction (AARE). For avoiding interferences when searching a ridge, an adaptive edge detection strategy is implemented. In addition, an adaptive core function constructed by using the signal characteristics keeps stability during peak exploration and curve continuity. This technique runs automatically because it does not require parameter tuning, minimizing user intervention.
	DCS	[22]	Fault diagnosis in rolling bearings through the variational mode decomposition (VMD) and degree of cyclostationarity (DCS) demodulation. At first place, the reconstruction of denoised signal is carried out by means of sparsity-based reconstruction factor. Next, fault characteristics are extracted through DCS, and finally, combined with VMD, the faults detection is performed.
	EMBM	[23]	Identification of defective components, case of the rolling bearings, on axial piston pumps. The fault detection is made through a tangent hyperbolic fuzzy entropy measure-based method that determines the most sensitive Wavelet packet transform (WPT).

**Table 2:** Fragment of the PQD classification according to IEEE-1159 standard [38].

PQD	Duration	Magnitude	Spectral Content
Transients—Oscillatory			
Low frequency	0.3–50 ms	0–4 pu ^1^	<5 kHz
Medium frequency	20 µs	0–8 pu	5–500 kHz
High frequency	5 µs	0–4 pu	0.5–5 MHz
Short duration variations—Instantaneous			
Sag	0.5–30 cycles	0.1–0.9 pu	
Swell	0.5–30 cycles	1.1–1.8 pu	
Short duration variations—Momentary			
Sag	30 cycles–3 s	0.1–0.9 pu	
Swell	30 cycles–3 s	1.1–1.4 pu	
Short duration variations—Temporary			
Sag	3 s–1 min	0.1–0.9 pu	
Swell	3 s–1 min	1.1–1.2 pu	
Voltage fluctuations			
Flicker	intermittent	0.1–7%	<25 Hz

^1^ The units are dimensionless; they are considered instead per unit (pu).

**Table 3:** Fragment of the power quality parameters according to IEC-61000-4-30 standard [39].

Parameters	Class	Measurement Method	Uncertainity	Measuring Range
Flicker	A	IEC 61000-4-15	IEC 61000-4-15	0.2~10.0 *P_st_*
	S	IEC 61000-4-15	Twice the permitted measurement uncertainty required by IEC 61000-4-15	0.4~4.0 *P_st_*
Dips and Swells	A	*U_rms(1/2)_*	Amplitude ±0.2% *U_din_* Duration +/− 1 cycle	N/A
	S	*U_rms_*_(1/2)_ on each measurement channel, or *U_rms_*_(1)_ on each measurement channel.	Amplitude ±1% of *U_din_* Duration +/− 1 cycle or +/− 2 cycles	N/A
Transient voltages IEC 61180	A	N/R	N/R	N/R
	S	N/R	N/R	N/R
Fast transients IEC 61000-4-4	A	N/R	N/R	N/R
	S	N/R	N/R	N/R

N/R = no requirement, N/A = not applicable, *U_din_* = value obtained from the declared supply voltage by a transducer ratio, *U_rms_*_(1/2)_ = value of the r.m.s., voltage measured over 1 cycle, commencing at a fundamental zero crossing, and refreshed each half-cycle, *U_rms_*_(1)_ = value of the r.m.s., voltage measured over 1 cycle and refreshed each cycle, *P_st_* = short-term flicker severity, unless otherwise specified, the evaluation time is 10 min, Subclasses advanced (A) and surveys (S) are considered, and subclass basic (B) will be removed in future from the standard.

**Table 4:** Techniques for ND and best hyperparameters used.

ND Technique	Hyperparameters	Description	Evaluated Values	Best Value
kNN	*k*	Number of the closest neighbors	3, 5, 7, 9, 11	3
OC-SVM	*v*	Regularization value	0.025, 0.03, 0.035, 0.04, 0.045	0.03
GMM	*RV*	Regularization value	0.0001, 0.001, 0.01, 0.1	0.01
SOM	*Q_re_*	Percentile for reconstruction error	90, 95, 98	90
IF	*n*	Structure dimension	8, 10, 12	8
SAE ^1^	*Q_u_*	Percentile of tree depth threshold for assigning the score of novelty	90, 95, 98	90

^1^ For SAE, a structure of 50-30-25 was configured for each case study, through several grid searches for the hyperparameters of L2W regularization, sparsity proportion, sparsity regularization, and epochs.

**Table 5:** Numerical results of the novelty detection obtained for the first case study of the SPG_DS.

ND Technique	Known (%)	Novelty (%)	Total Performance (%)
SAE	88	100	94
OC-SVM	98.5	100	99.25
kNN	94.5	100	97.25
GMM	78	100	89
SOM	96.5	100	98.25
IF	96	100	98

**Table 6:** Numerical results of the novelty detection obtained for the second case study of the SPG_DS.

ND Technique	Known (%)	Novelty (%)	Total Performance (%)
SAE	87	100	93.5
OC-SVM	98.5	100	99.25
kNN	94.5	100	97.25
GMM	81.5	100	90.75
SOM	95.5	100	97.75
IF	96	100	98

**Table 7:** Numerical results of the novelty detection obtained for the third case study of the SPG_DS.

ND Technique	Known (%)	Novelty (%)	Total Performance (%)
SAE	95	100	97.5
OC-SVM	79.5	100	89.75
kNN	93.5	95	94.25
GMM	66	98.5	82.25
SOM	99	15	57
IF	97.5	86.5	89.5

**Table 8:** Numerical results of the novelty detection obtained for the fourth case study of the SPG_DS.

ND Technique	Known (%)	Novelty (%)	Total Performance (%)
SAE	90.5	100	92.25
OC-SVM	83.5	100	91.75
kNN	91.5	100	95.75
GMM	77	100	88.5
SOM	91	69	77.5
IF	97	80	88.5

**Table 9:** Numerical results of the novelty detection obtained for the fifth case study of the SPG_DS.

ND Technique	Known (%)	Novelty (%)	Total Performance (%)
SAE	87.5	100	93.75
OC-SVM	99	100	99.5
kNN	94	100	97
GMM	82.5	100	91.25
SOM	96.5	90	93.25
IF	96.5	100	98.25

**Table 10:** Numerical results of the novelty detection obtained for the first case study of the WPG_DS.

ND Technique	Known (%)	Novelty (%)	Total Performance (%)
SAE	87.33	100	93.68
OC-SVM	98.33	100	99.18
kNN	93.66	100	96.85
GMM	80.68	100	90.33
SOM	96	75.01	85.5
IF	93.66	100	96.84

**Table 11:** Numerical results of the novelty detection obtained for the second case study of the WPG_DS.

ND Technique	Known (%)	Novelty (%)	Total Performance (%)
SAE	85.01	100	92.5
OC-SVM	100	100	100
kNN	94.33	100	97.18
GMM	80.33	100	90.17
SOM	97.33	76.67	87.01
IF	95.33	100	97.68

**Table 12:** Numerical results of the novelty detection obtained for the third case study of the WPG_DS.

ND Technique	Known (%)	Novelty (%)	Total Performance (%)
SAE	87.34	100	93.66
OC-SVM	69.68	100	84.83
kNN	91.67	100	95.83
GMM	74.34	98.34	86.34
SOM	92.65	80.01	86.35
IF	94.66	81.67	88.18

**Table 13:** Numerical results of the novelty detection obtained for the fourth case study of the WPG_DS.

ND Technique	Known (%)	Novelty (%)	Total Performance (%)
SAE	88	100	94
OC-SVM	64.33	100	82.17
kNN	91	100	95.5
GMM	83.66	86.67	85.18
SOM	95	25.34	60.16
IF	96.66	47.67	72.17

**Table 14:** Numerical results of the novelty detection obtained for the fifth case study of the WPG_DS.

ND Technique	Known (%)	Novelty (%)	Total Performance (%)
SAE	82.33	100	91.18
OC-SVM	99.33	100	99.67
kNN	91.99	100	96.01
GMM	80.67	100	90.33
SOM	92.33	75.34	83.84
IF	93.67	100	96.84

## Data Availability

Not applicable.

## Appendix A

The 20 statistical and non-statistical indicators used in the comparative study and computed in the time domain, frequency domain, and time—frequency domain can be obtained through Expressions (A1) to (A20) in Table A1.

**Table A1:** Statistical and non-statistical indicators.

Indicator	Equation	
Mean	$\bar{x}=\frac{1}{n}\cdot \overset{n}{\underset{i=1}{\sum }}(x_{i})$	(A1)
Maximum Value	$\hat{x}=\max\left(x\right)$	(A2)
Root Mean Square (RMS)	$RMS=\sqrt{\frac{1}{n}\cdot \overset{n}{\underset{i=1}{\sum }}{\left(x_{i}\right)}^{2}}$	(A3)
Square Root Mean (SRM)	$SRM={\left(\frac{1}{n}\cdot \overset{n}{\underset{i=1}{\sum }}\sqrt{\left|(x_{i}\right|}\right)}^{2}$	(A4)
Standard Deviation	$\sigma =\sqrt{\frac{1}{n}\cdot \overset{n}{\underset{i=1}{\sum }}{\left(x_{i}-\bar{x}\right)}^{2}}$	(A5)
Variance	$\sigma^{2}=\frac{1}{n}\cdot \overset{n}{\underset{i=1}{\sum }}{\left(x_{i}-\bar{x}\right)}^{2}$	(A6)
RMS with shape factor	$SF_{RMS}=\frac{RMS}{\frac{1}{n}\cdot \sum_{i=1}^{n}\left|x_{i}\right|}$	(A7)
SRM with shape factor	$SF_{SRM}=\frac{SRM}{\frac{1}{n}\cdot \sum_{i=1}^{n}\left|x_{i}\right|}$	(A8)
Crest Factor	$CF=\frac{\hat{x}}{RMS}$	(A9)
Latitude Factor	$LF=\frac{\hat{x}}{SRM}$	(A10)
Impulse Factor	$IF=\frac{\hat{x}}{\frac{1}{n}\cdot \sum_{i=1}^{n}\left|x_{i}\right|}$	(A11)
Skewness	$S_{k}=\frac{E\left[{(x_{i}-\bar{x})}^{3}\right]}{\sigma^{3}}$	(A12)
Kurtosis	$k=\frac{E\left[{(x_{i}-\bar{x})}^{4}\right]}{\sigma^{4}}$	(A13)
5th Moment	$5th_{M}=\frac{E\left[{(x_{i}-\bar{x})}^{5}\right]}{\sigma^{5}}$	(A14)
6th Moment	$6th_{M}=\frac{E\left[{(x_{i}-\bar{x})}^{6}\right]}{\sigma^{6}}$	(A15)
Energy	$E=\overset{n}{\underset{i=1}{\sum }}\left({\left|x_{i}\right|}^{2}\right)$	(A16)
Entropy	$ET=-\overset{n}{\underset{i=1}{\sum }}x_{i}{}^{2}\log\left(x_{i}{}^{2}\right)$	(A17)
Range	$R=Max\left(x_{i}\right)-Min\left(x_{i}\right)$	(A18)
Form factor	$FF=\frac{\bar{x_{i}}}{RMS_{i}}$	(A19)
Log energy Entropy	$LE=-\overset{n}{\underset{i=1}{\sum }}\log\left(x_{i}{}^{2}\right)$	(A20)

From this table, *x* is the input data vector from which the statistical features are extracted; *n* is the total amount of data in the sample set; *i* is the corresponding *ith* sample that takes values from i=1,2,3,…,n.
